# Formulation and Characterization of Fe_3_O_4_@PEG Nanoparticles Loaded Sorafenib; Molecular Studies and Evaluation of Cytotoxicity in Liver Cancer Cell Lines

**DOI:** 10.3390/polym15040971

**Published:** 2023-02-16

**Authors:** Mona Ebadi, Ahmad Rifqi Md Zain, Tengku Hasnan Tengku Abdul Aziz, Hossein Mohammadi, Clarence Augustine TH Tee, Muhammad Rahimi Yusop

**Affiliations:** 1 College of Physics and Electrical Information Engineering, Zhejiang Normal University, Jinhua 321017, China; 2 Department of Chemical Sciences, Faculty of Science and Technology, Universiti Kebangsaan Malaysia (UKM), Bangi 43600, Selangor, Malaysia; 3 Materials Synthesis and Characterization Laboratory, Institute of Advanced Technology (ITMA), Universiti Putra Malaysia, Serdang 43400, Selangor, Malaysia; 4 Institute of Microengineering and Nanoelectronics (IMEN), Universiti Kebangsaan Malaysia (UKM), Bangi 43600, Selangor, Malaysia; 5 Faculty of Mechanical Engineering, Universiti Teknologi Malaysia, Johor Bahru 81310, Johor, Malaysia

**Keywords:** polyethylene glycol, sorafenib, iron oxide nanoparticles, cancer cells

## Abstract

Iron oxide nanoparticles are one of the nanocarriers that are suitable for novel drug delivery systems due to low toxicity, biocompatibility, loading capacity, and controlled drug delivery to cancer cells. The purpose of the present study is the synthesis of coated iron oxide nanoparticles for the delivery of sorafenib (SFB) and its effects on cancer cells. In this study, Fe_3_O_4_ nanoparticles were synthesized by the co-precipitation method, and then sorafenib was loaded onto PEG@Fe_3_O_4_ nanoparticles. FTIR was used to ensure polyethylene glycol (PEG) binding to nanoparticles and loading the drug onto the nanoshells. A comparison of the mean size and the crystalline structure of nanoparticles was performed by TEM, DLS, and X-ray diffraction patterns. Then, cell viability was obtained by the MTT assay for 3T3 and HepG2 cell lines. According to FT-IR results, the presence of O–H and C–H bands at 3427 cm^–1^ and 1420 cm^–1^ peak correlate with PEG binding to nanoparticles. XRD pattern showed the cubic spinel structure of trapped magnetite nanoparticles carrying medium. The magnetic properties of nanoparticles were examined by a vibrating-sample magnetometer (VSM). IC_50_ values at 72 h for treatment with carriers of Fe_3_O_4_@PEG nanoparticle for the HepG2 cell line was 15.78 μg/mL (*p* < 0.05). This study showed that Fe_3_O_4_ nanoparticles coated by polyethylene glycol and using them in the drug delivery process could be beneficial for increasing the effect of sorafenib on cancer cells.

## 1. Introduction

Cancer is one of the most lethal diseases in human history. Liver cancer is the fourth most common cancer in the world, which leads to significant morbidity, particularly in southeastern countries. Chemotherapy has been used as one of the ordinary approaches to treat cancer diseases and enhance the patient’s life span [1]. The side effects of drugs for chemotherapy can significantly decrease the results of treatment and restrict their usage. The success of chemotherapy depends on the delivery of sufficient drug concentration to the tumor cells without being cytotoxic to the healthy cells in the patient’s body [2].

One of the most popularly used drugs for chemotherapy in cancer treatment is Sorafenib (SFB) [3]. Several studies have confirmed its high effectiveness in vitro and in vivo. Sorafenib connects to DNA and impedes the formation of nucleic acid, and this leads to impairment of molecular structure and further steric effects [4]. As a consequence, the growth and proliferation of cancer cells in the body are impeded. Nonetheless, the direct injection of SFB can be significantly harmful due to its high toxicity, low stability in the bloodstream, difficult infiltration into the cancer tissues, reduced drug performance by decomposing enzymes in the body, and adverse side effects by infiltration into the healthy parts. The side effects of drugs for chemotherapy resembling SFB can significantly decrease the results of treatment and restrict their usage [5]. Thus, the design of smart drugs is a major issue in novel drug delivery systems. The purpose of developing novel drug delivery system include sustained drug release, maintenance of drug concentration in the therapeutic range for a suitable time interval, and specific drug transport to the specific tissue [6]. Generally, the drug delivery systems are referred to as carriers that have the ability to connect to a drug, encapsulate the drug and carry the drug in the body. The nanoparticles loaded with anticancer drugs are a promising approach to target cancer cells. Drug delivery systems based on nanoparticles have been found to alter the drug pharmaceutics, increase the drug half-life, and increase the drug permanence in the bloodstream, which led to a significant increase in the efficacy of drug treatment. This targeted delivery system reduces the dosage and the toxicity of drugs to healthy tissues [2,7,8].

Most previous research has focused on increasing the efficiency of the anticancer drug through specific drug delivery to tumors. However, conventional chemotherapy may not be successful in some of the above-mentioned conditions as the anticancer drugs are inclined to disperse the whole body and kill all the cells. Additionally, this system has better infusibility in human cells depending on their size, which allows drug delivery through intravenous and subcutaneous injection or other routes [2]. The drug delivery using small particles allows faster dissolution in the bloodstream, which leads to targeted drug delivery in specific cells and tissues. Long-term treatment approaches such as chemotherapy can cause significant patient dissatisfaction. Thus, chemotherapy using drug-containing nanoparticles can cause more comfort to the patient and be more effective by eliminating daily injections [9,10]. 

In this regard, different structures have been developed for drug delivery systems, including dendrimers, micelles, liposomes, and polymer-based magnetic nanoparticles [7,11]. Magnetite nanoparticles (Fe_3_O_4_) are a suitable candidate to be used as smart nanocarriers due its characteristics such as wide active surface, suitable size and distribution to pass through the blood vessels, high biocompatibility, high capacity for drug loading, low biotoxicity, and magnetic properties into the target tissue. Additionally, magnetic nanoparticles of Fe_3_O_4_ nanoparticles can enhance the delivery and efficacy of anticancer drugs. Thus, they play a key role in transporting the drugs to the tumor tissues [12,13,14]. 

Among diverse techniques developed for magnetite synthesis, we could mention the co-precipitation method. Co-precipitation is an important procedure and the most widely used for the synthesis of Fe_3_O_4_ nanoparticles. It is spotlighted as a simple, fast process, cost-effective, eco-friendly route, high yield, high product purity, and easily reproducible for various applications [15,16]. One of the main advantages of this method is that it can directly obtain water-soluble nanoparticles without requiring hazardous organic solvents or treatments under high pressure or temperature. However, the properties of the obtained particles, such as size, shape, and composition, are highly dependent on the reaction parameters (temperature, pH, and type of basic solution) [17]. In addition, Fe_3_O_4_ nanoparticles obtained in this way are often not stable and hence are stabilized by using surfactants or functionalized polymers. Many commercial Fe_3_O_4_ nanoparticles which are used in medicine, targeted drug delivery, and biosensing are produced by this method. In addition, this technique can also be used to prepare water-dispersible Fe_3_O_4_ nanoparticles and form a durable suspension for several days [18]. M. Mahmoodi et al. have used this method for the synthesis of Fe_3_O_4_ nanoparticles to strengthen the microwave-absorbing performance of the porous carbon [19].

Recently, magnetic hyperthermia (MHT) accompanied by chemotherapy has been proven to be a promising route to remedy tumors and even cancer in human bodies [20]. One of the significant features of Fe_3_O_4_ nanoparticles, which makes them appropriate for medical applications, such as MHT of tumor cells, is their magnetic tracer and heat generation capability under an alternating current (AC) magnetic field. Since cancerous cells are more sensitive to heat than healthy cells and perish at temperatures above 42 °C [21]. The magnetic nanoparticles are also dispersed in non-magnetic liquids, such as water, and then are injected into the tumor area of the patient [20]. Jalili et al. [22] have prepared CoFe_2_O_4_/Fe_3_O_4_ nanocomposites for MHT application. They have reported that CoFe_2_O_4_/Fe_3_O_4_ nanocomposites can be useful in various applications.

Tracking the cells using magnetic nanoparticles, which are observable by magnetic resonance imaging (MRI), offers a new approach to empirical observations of cellular treatments. A combination of these two nanoparticles can be synthesized via a single-stage process using the precipitation of alkaline iron salts comprising ferrous and ferric. Different compounds exist in the human body, which contain 3 to 4 g of iron, including protein, transferring, hemosiderin as well as hemoglobin [23,24]. When the magnetic nanoparticles are decomposed in the human body, significant amounts of iron enter the exiting iron storage in the body and adjust the amount of iron there. The medical dosage for the human body is probably less than a milligram [25]. At room temperature, they show superparamagnetic behavior. Superparamagnetic materials are materials with a single magnetic domain, and all their atomic spines are uniformly magnetized in a similar direction. In other words, they become significantly magnetized under a magnetic field that is not constant and disappear by the elimination of the magnetic field. The Fe_3_O_4_ nanoparticles under a magnetic field can carry the therapeutic agents and modify the drug delivery capacity without pathway deviance in the body using this magnetic behavior [26].

Polymeric coatings can be used as a coating on Fe_3_O_4_ nanoparticles to increase their biocompatibility. Polymeric coating not only increases their colloidal stability but also enhances their durability in the bloodstream. Similar to this research study, Jacob McCright et al. argued that coating nanoparticles with polyethylene glycol (PEG)-polypropylene oxide copolymers could enhance the accumulation of nanoparticles and their stability after intradermal administration [27]. In another study, Lin et al. investigated the stability of different drug carriers using cross-linked micelle based on poly (carboxybetaine methacrylate), poly(ε-caprolactone), and poly(S-2-hydroxyethyl-O-ethyl dithiocarbonate methacrylate) [28]. This study found that micelles possessed excellent colloidal stability. This is because the connection and surface absorption of proteins on the surface of Fe_3_O_4_ nanoparticles is impeded. In this regard, different types of natural and synthetic polymers, such as albumin, PEG, dextran, chitosan, and poloxamer, have been used to enhance the magnetite nanoparticles’ efficiency in biological systems further [29]. For instance, in the research conducted by Tian et al. in 2019, PEG was used as a coating on nanoparticles to deliver DOX [30]. In another study conducted by Jang et al. in 2015, the polyarabic acid polymer was used as a coating on nanoparticles for SFB drug delivery and introduced the synthesized system as theranostics [31]. The selection of polymer directly depends on biocompatibility, hydrophilicity, and lack of protein absorption.

PEG is a polymer that has recently been a topic of research interest. Higher biocompatibility of PEG than previously used polymers, together with other characteristics such as low viscosity and lack of protein absorption on their surface, make this polymer a suitable candidate for in vivo applications. The PEG prevents hydrophobic and electrostatic interactions. The coating of magnetic nanoparticles with PEG can act as a place for drug loading [32]. The structure of this polymer is comprised of hydrophilic and hydrophobic groups, and consequently, they can carry hydrophilic and hydrophobic therapeutics. The presence of many hydroxyl groups on the surface of this polymer increases the hydrophilicity of coated nanoparticles and incorporates the connection of tracking and targeting agents. The presence of such coatings assists in the durability of nanoparticles in the biological fluids and bloodstream, the reduction in toxic effects, and sustained drug release to the cells [33]. 

The drug-containing magnetic nanoparticles enter the body through intravenous or intramuscular injection or using a tablet and are transferred to the target cells using an external magnetic field. The positive advantage of drug magnetic nanoparticles is that they can easily identify by the cancer cell receptors. They can cross the intestinal mucus layer and reach the bloodstream. Generally, the drug release mechanism is performed in the cells as follows: 

The drug-containing magnetic nanoparticles can enter the cells by bypassing the negatively charged cell membrane. Inside the cells, bonds between drug and polymer will be destroyed by lysosomes, and therefore the drugs can be released [34,35]. 

In the present study, this was performed experimentally; Fe_3_O_4_ nanoparticles were synthesized by the co-precipitation method. In this process, the dry nitrogen atmosphere is utilized to protect iron salts from oxidation. In addition, PEG with suitable biocompatibility was used as a coating agent, and the SFB drug was loaded on the nanocarrier. After the synthesis, the Fe_3_O_4_ particles were characterized by different techniques. Furthermore, the magnetism of Fe_3_O_4_ particles was measured and compared with and without coating under a magnetic field. In addition, the effect of biotoxicity on the normal human fibroblast (3T3) and hepatocellular carcinoma (HepG2) cell lines via transporting SFB in both free and embedded conditions with Fe_3_O_4_@PEG was evaluated. In the previous studies, polymer-based nanoparticles carrying SFB have been investigated; however, their nanoparticle synthesis and loading routes were different from our research. The in vitro drug release from this delivery system showed that it could be a suitable system to carry SFB anticancer drugs and that the sustained release of drugs within a predetermined time interval can be more effective against cancer.

## 2. Methods and Methods

### 2.1. Experimental 

In this study, Fe_3_O_4_ nanoparticles were synthesized by the chemical co-precipitation from the solution containing iron salts in the alkaline medium under N_2_ gas and room temperature. For this purpose, first, a solution containing 1.28 mols ferric chloride hexahydrate and 0.64 mols ferrous chloride tetrahydrate as a source of iron was prepared by dissolving it in double distilled water under a high-speed mechanical stirrer. Another solution of sodium hydroxide [NaOH>99%] was produced and used as a source of alkalinity. The alkaline source was added dropwise to the iron source, and the magnetic stirring at 700 rpm lasted for 30 min. Nitrogen gas also flowed from the reaction medium in a closed system during the synthesis process. The precipitated powder was separated from the solution by applying an external magnetic field, and the upper liquid was slowly drained. Then the resulting powder was washed twice with distilled water and placed in a centrifuge for 15 min. As surface coating of Fe_3_O_4_ nanoparticles, 4 gr of PEG was added to 100 mL distilled water and then mixed with Fe_3_O_4_ precipitates, and this solution was placed in an autoclave for 24 h at a temperature of 200 °C and then washed and centrifuged. This was repeated to remove the excess polymer. To load SFB anticancer liver drug by Fe_3_O_4_@PEG nanoparticles, the nanocomposite of Fe_3_O_4_@PEG was combined with 3 gr of SFB, which was dissolved in dimethyl sulfoxide (DMSO) (Merk, Darmstadt, Germany). They mixed for one night using rapid centrifugation. Then Fe_3_O_4_@PEG/SFB nanoparticles were washed with sterile water and dried by freeze dryer.

### 2.2. Materials 

Iron(III) chloride hexahydrate, Iron(II) chloride tetrahydrate with 99% purity, and sodium hydroxide (99% purity) for synthesized magnetite nanoparticles were provided from Merck (Darmstadt, Germany). Polyethylene glycol (PEG 6000, 98%) was purchased from Organics Company (Coventry, England). Polydispersity for PEG with a molecular weight of 6K is about 1.04, calculated using the equation ĐM = Mw/Mn, where Mw is the weight-average molar mass, and Mn is the number-average molar mass. Sorafenib, as the primary therapeutic, was sourced from Xi’an Yiyang Bio-Tech company, Xi’an, China, at 98.5% purity. Methanol HPLC grade and ethanol HPLC grade with high purity were acquired from Sigma Aldrich company (Burlington, MA, USA). In addition, the required laboratory-grade chemicals were used.

### 2.3. Instrumentation

The crystallinity and structure of the nanocomposite were assessed by X-ray diffraction (XRD-6000 with Cu K𝛼 radiation at 40 kV/30 mA, in the range of angles 2° < 2θ < 80°, Shimadzu, Japan). Fourier-transform infrared spectroscopy (FTIR) technique was used to determine the magnetic core, polymer, and drug (Thermo Nicolet 6700, optic resolution 0.09 cm^–1^, be configured for spectral ranges of 500–4000 cm^–1^, Madison WI, USA). The shape, size, and size distribution of the nanocomposite were observed by transmission electron microscopy instrument (TEM, Hitachi H-7100, Tokyo, Japan). Magnetic properties were analyzed using a vibrating sample magnetometer (VSM, the Lake Shore Cryotronics model 7404, Westerville, OH, USA) in the range of high and low field intensity (field intensity −10,000 to 10,000 Oersted) to measure the magnetism of the synthesized samples. The thermal stability of materials has been investigated by the thermogravimetric/differential thermogravimetric analysis (TGA/DTG) method (Mettler-Toledo model TG/DTA, Greifensee, Switzerland). TGA traces at the heating rate of 10 °C.min^–1^ in the range of 20–1000 °C. The profile of SFB drug release in phosphate-buffered solution (PBS) media at pH similar to human plasma (7.4) and cancerous areas (4.8) was obtained by Ultraviolet-visible spectroscopy (UV-Vis, Perkin Elmer Lambda 35, Mundelein, Illinois, USA) in the time intervals of 6 h for 4 days (96 h). 

### 2.4. Quantitative Evaluation of Cytotoxicity Effect by Colorimetric MTT Assay

Culture medium RPMI 1640 (Roswell Park Memorial Institute medium) (Merk, Darmstadt, Germany), trypsin, M-EDTA, penicillin (100/ units/mL), and streptomycin (100 mg/mL) as antibiotics, are used in cell culture test, and all were purchased Nacalai Tesque (Kyoto, Japan). Viable cells were assessed using the 3-[4,5-dimethylthiazol-2-yl]-2,5-diphenyltetrazolium bromide (MTT) and fetal bovine serum (FBS) (Sigma-Aldrich, Burlington, MA, USA). HepG2, human liver cancer cells, and normal human fibroblast, 3T3 cell lines were bought from the cell bank located at the National Center of Genetic Engineering and Biotechnology (Pathum Thani, Thailand). Deionized water was used in all the experiments.

In the second step, the cellular metabolic activity was assessed by the tetrazolium-based MTT assay. Solutions containing 0.1% DMSO and RPMI (1:1) were prepared, and the cells were treated with sorafenib (SFB) and nanocomposite. As part of the toxicity evaluation of synthesized NPs, cell-based in vitro assays, in environs of HepG2 and 3T3 cell lines, the two cell types, liver cancer cells and normal fibroblast cells were grown in a cell culture medium, and PRMI along with 10% FBS and 1% antibiotics (10,000 μg/ mL of streptomycin and 10,000 units/mL of penicillin) and were incubated at a temperature of 37 degrees Celsius in an incubator 5% CO_2_ 95% with RH = 100. To perform further analysis, to achieve at least 80% confluence, they were harvested from the bottom of the flask by 1m M-EDTA and 0.25% trypsin. Cell sedimentation was transferred and seeded in 96-well tissue culture plates. After 24 h placed in an incubator, the cells were obtained in different concentrations (from 1.25–100 μg/mL) in the same medium. The nanoparticles were placed in the well plates until the final volume of 100 μL and kept in an incubator for one night. In the second step, measuring the number of cells by the colorimetric method, an MTT test was performed for both cell lines in separate plates. The cell suspension was prepared from both cell lines, and 10 μL of the 5 mg/mL MTT was added to each plate. The cells were incubated for 3 h. The purple formazan salt was dissolved in DMSO and added to each plate. After the dye particles were well dissolved, the light absorbance at a 570 nm wavelength by a microplate reader (Biotek LE800, Winooski, VT, USA) was read.

## 3. Results and Discussions

### 3.1. XRD Analysis

**Figure 1:**
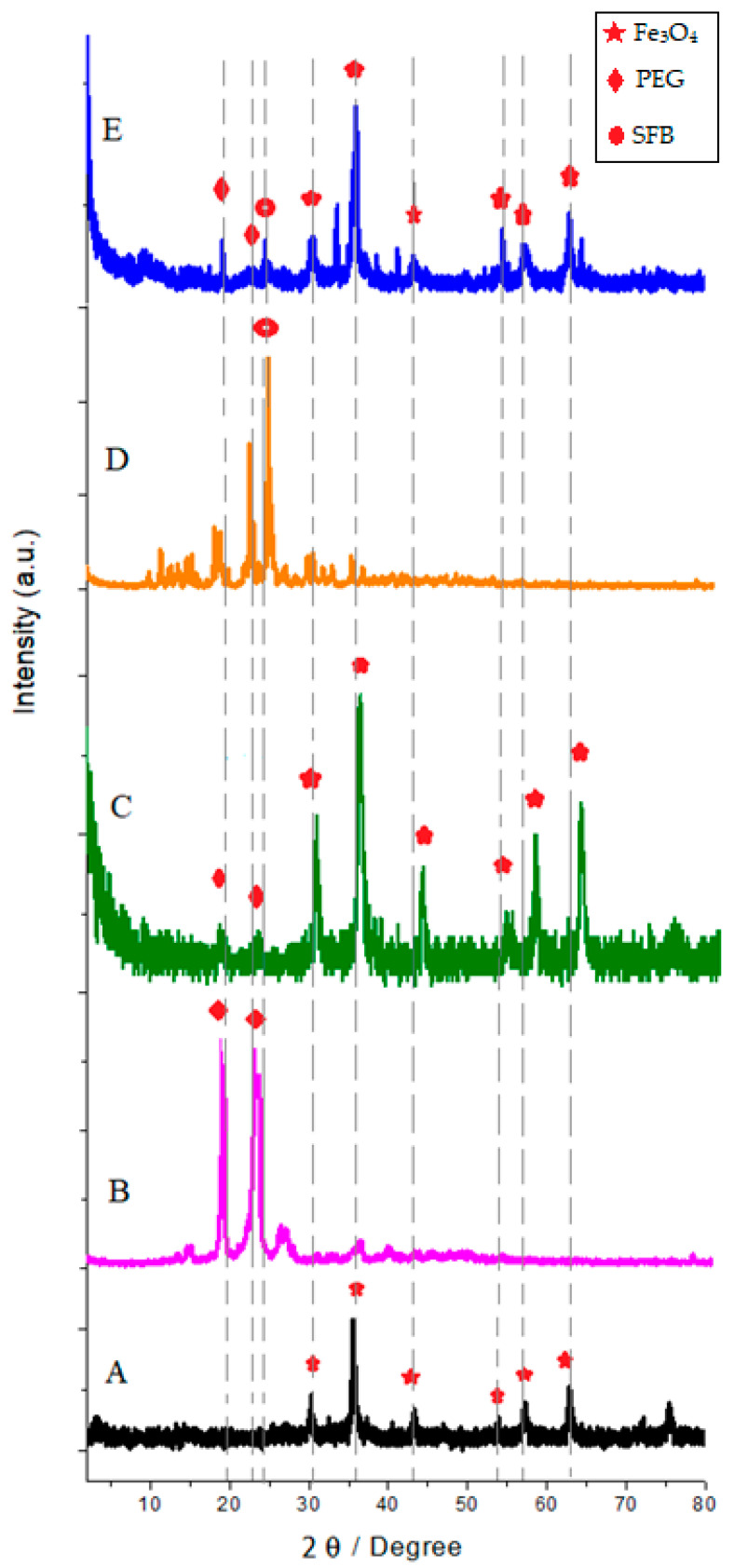
XRD of powdered (**A**) Fe_3_O_4_ nanoparticles; (**B**) pure PEG; (**C**) Fe_3_O_4_@PEG; (**D**) SFB, (**E**) Fe_3_O_4_@-PEG_SFB.

Figure 1 shows the XRD pattern of the samples at room temperature. Figure 1A indicated the formation of the Fe_3_O_4_ crystalline structure and confirmed the inverse spinel structure. These patterns reveal that the dominating phase was related to the Fe_3_O_4_ nanoparticles with a spinel cubic structure and all the reflections match with the Joint Committee on Powder Diffraction Standard (JCPDS) reference database No. 86-2267 [36,37,38,39]. 

The XRD peaks ascertained that the peaks in 19.3°, 23.5°, and between 10–35° can be attributed to SFB and PEG, respectively [40,41,42]. The peaks of the final sample (Figure 1E) showed that the main composition of nanoparticles was Fe_3_O_4,_ and no prominent other extra peaks in the XRD pattern were present as impurities. Therefore, this gives clear evidence for the presence of Fe_3_O_4_ nanoparticles in the synthesized sample. 

There was no significant difference between the nanoparticle patterns A, B, C, and D with the ultimate sample (E). Only the intensities of raw particle’s peaks due to the surface layer on the particles were decreased. In addition, it was observed that the peak width in pattern E was reduced by the effect of PEG and SFB. This indicates an increase in the size of the crystals. Additionally, the lowest peak intensities were related to pattern E, which is associated with magnetic drug carriers confirming successful drug loading. Interestingly, the positions of the peaks in the XRD pattern of Fe_3_O_4_@PEG_SFB prepared by the addition of polymer and drug are unaltered. The polymer likely acts, in this case, as a coating agent. The XRD intensities of PEG alone are higher than those of composite (Fe_3_O_4_@PEG). It seems that a decrease in the crystallite size of PEG has occurred in the composites. Additionally, only PEG and SFB peaks were observed in the case of the Fe_3_O_4_@PEG_SFB composite sample. It indicates that no chemical interaction has occurred between PEG and Fe_3_O_4_ or the drug. Figure 1D exhibits the XRD pattern of net SFB with sharp reflection at 2θ = 25°and and is highly crystalline. In sample E, the reflection patterns of SFB with slight left shifting compared to the free SFB confirmed the loading of SFB onto the final composite.

### 3.2. FT-IR Analysis

**Figure 2:**
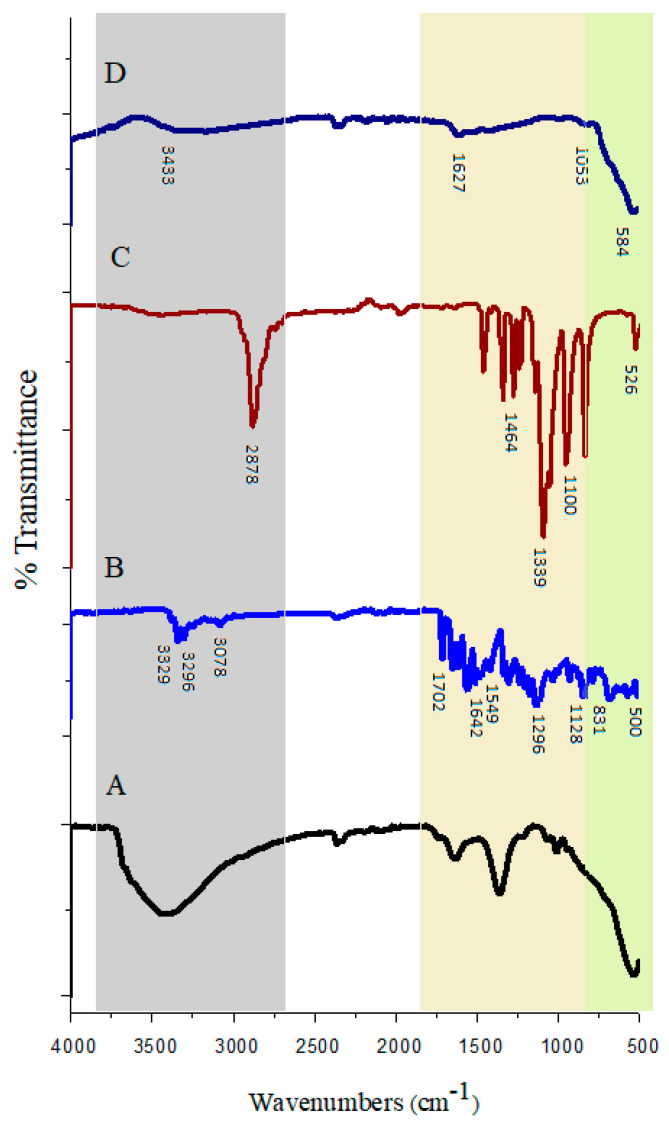
FTIR spectra for (**A**) Fe_3_O_4_@−PEG-SFB; (**B**) SFB; (**C**) pure PEG; (**D**) Fe_3_O_4_ nanoparticles.

Figure 2 shows the FT-IR spectra of nanoparticles with and without coating to corroborate the structure of the products. Figure 2D depicts the FT-IR spectra of pure Fe_3_O_4_ in which two peaks (absorption band) are observable. The wide absorption band was in the range of 2500–3600 cm−^1,^ which is ascribed to the strong stretching vibrations of hydrogen bonds with hydroxy groups (OH) absorbed into the samples from the environment. The second absorption band was found to be at 584 cm−^1,^ which is attributed to the vibrating bond of Fe–O in the Fe_3_O_4_. This further indicates that iron (Fe) is the key structural element in the compound [43]. These shifted bands in the latest synthesized sample (Fe_3_O_4_@-PEG_SFB) to the lower wavenumbers confirmed the presence of Fe_3_O_4_.

Figure 2C shows the FTIR spectra of PEG. For the pure PEG sample spectrum, the 2878 cm−^1^ peak is due to aliphatic C–H stretching vibrations in the PEG chain. The peaks at 1464 cm^–1^ and 1339 cm−^1^ are due to C–H bending vibrations. The O–H and C–O–C stretching vibrations produce peaks at 1278 and 1095 cm−^1^, respectively [44]. In Figure 2B for the drug sample, the peaks at 3329 cm−^1^ and 3296 cm−^1^ are related to the N–H stretching of amide. Additionally, the measured peak corresponds to the aromatic C–H stretching band, and the amide group was found at a wavelength of 3078 cm−^1^ and 1642 cm−^1^, respectively [45]. 

The FTIR spectra of nanoparticles coated with PEG and targeted with SFB showed wide vibrating peaks in the range of 3000–3600 cm−^1,^ which were ascribed to the presence of O–H. (Figure 2A). The increase in peak intensity of O–H in Figure 2A indicates not only the polymerization but also an increase in the hydrophilicity of coated nanoparticles. In the region near 1600 cm−^1^, the stretching band of H–O–H is recorded, which revealed that nanocomposite surfaces readily absorb H_2_O molecules when exposed to the atmosphere.

Furthermore, the comparison between the nanoparticles with and without coating showed the presence of fairly obvious peaks at 1050–1100 cm−^1^ is attributed to the vibrations of C–O bonds. This demonstrated that some of the PEG molecules had been successfully connected to the surface of Fe_3_O_4_ nanoparticles. The presence of PEG coating on the nanoparticles led to the absorption of irradiation by the core of Fe_3_O_4_. The peaks for the nanoparticles with PEG coating was found to be weaker than pure nanoparticle. Therefore, the FTIR spectra showed the existence of van der Waals interactions between the chain of PEG and Fe_3_O_4_ nanoparticles in the polymeric media. As can be observed, the lack of significant difference between FTIR spectra of the nanocomposite sample and PEG, together with high absorption of the C–H bonds related to PEG in the Fe_3_O_4_@-PEG_SFB sample, revealed that the surface of the Fe_3_O_4_ nanoparticles in the final sample was fully covered by PEG. The intensity of these peaks was increased significantly in the pure samples compared to the coated sample. In addition, the peaks for drug-containing PEG Fe_3_O_4_ nanoparticles were weaker than both of them. This indicated drug loading between the layers of PEG and around Fe_3_O_4_ nanoparticles. There are no additional peaks nor shifts of the individual peaks in the spectrum of the nanocomposite. In the FTIR spectra, the bending and stretching of bonds were confirmed.

### 3.3. Magnetic Properties

**Figure 3:**
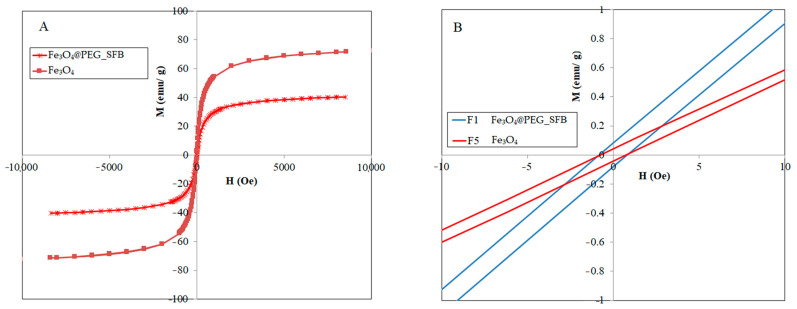
(**A**) Magnetic properties; (**B**) Hysteresis curve in fields close to zero of synthesized Fe_3_O_4_ particles and Fe_3_O_4_@−PEG_SFB nanocomposite.

Figure 3 shows the magnetic hysteresis curve, which was obtained from the powder of the samples. It is clear from this figure that at room temperature, the magnetic hysteresis is almost negligible, and because the value of M did not reach a constant limit, the remanent magnetization value in the obtained results was low, so samples showed a behavior similar to the superparamagnetic characteristic. This became obvious by the transformation of the hysteresis ring to an S-shaped curve. Because the PEG-coated samples are less than 25 nm, thus, all the samples are superparamagnetic, further confirmed by the negligible coercivity. This was also the reason for the superparamagnetization of magnetite nanoparticles. It should be noted that the superparamagnetic behavior shown by the nanoparticle means that due to the reduction in the particle size to the nano level (as confirmed by TEM micrographs), the magnetic domains in the particle were oriented towards one region, which is referred to as sing-domains material.

The magnetic saturation for the Fe_3_O_4_ nanoparticles was estimated to be 73 emu/g. However, this value was significantly decreased to 37 emu/g for the Fe_3_O_4_@PEG_SFB nanocomposite. The reduction in Fe_3_O_4_ saturation magnetization (Ms) may be a result of the presence of a non-magnetic layer of polymer and drug on the particle surface. Tomitaka et al. [46] have compared the effect of double-layer and single-layer surface activators on the stability of the ferrofluid containing Fe_3_O_4_ nanoparticles and observed that magnetization reduced with the increase in surface activator layers, which is the reason for the reduction in the number of magnetic particles by increasing the amount of surface activator. Therefore, it can be concluded that the formation of an additional layer on the surface of Fe_3_O_4_ nanoparticles reduces magnetic penetration and magnetic properties.

According to these data, the Ms value of pure Fe_3_O_4_ nanoparticles in this research was estimated to be 73 emu/g which is less than that for the bulk sample (93 emu/g). These Ms data indicated an increase in effective magnetic anisotropy with a decrease in particle size, as expected from the increased surface/volume ratio in small magnetic nanoparticles. This decrease in the Ms values with a decrease in size can be explained by a magnetically disordered surface layer and the existence of a spin-disordered layer at the particle surface, which is magnetically dead. When the size of Fe_3_O_4_ particles is reduced to the nanometer scale, the surface effects become progressively more important. This can be attributed to the increase in the relation between the number of atoms that constitute the surface region to those in the bulk. In magnetic materials such as Fe_3_O_4_, the magnetic order in the surface of the particles is modified, and different properties, such as magnetization, may be strongly affected. In ferromagnetic systems, the magnetic coupling in the surface region can become highly frustrated, leading to a depletion of ferromagnetism. For this reason, this surface layer is usually named the magnetic dead layer (MDL). The presence of a magnetic dead layer is usually supposed to cause a degradation of magnetism due to highly frustrated spin configurations [47,48,49,50].

To further investigate the magnetic properties of the particles and to observe the superparamagnetic property, the hysteresis curve of the particles was measured by VSM in fields around zero Orsted (–10 to 10 Orsted), which is shown in Figure 3B. As can be seen, the magnetic curves were in the form of a straight line and passed through the origin, so the coercive field and remanent magnetization exhibit immeasurable values, which again confirms the superparamagnetic properties of particles upon the application of the magnetic field.

Meanwhile, as the thickness of the layer coating layers increases due to the increase in the negative charge on the surface of the nanoparticles and coulomb repulsion, the dispersion of nanoparticles also increases, but the magnetic property decreases. This phenomenon occurs due to the diamagnetic property of the polymer shell around the magnetic nanoparticles and also the increase in the overall mass compared to the magnetic material. Despite the reduction in Ms values, the magnetic property of these nanoparticles is still sufficient for use in biological applications.

### 3.4. TGA/DTG Analysis

**Figure 4:**
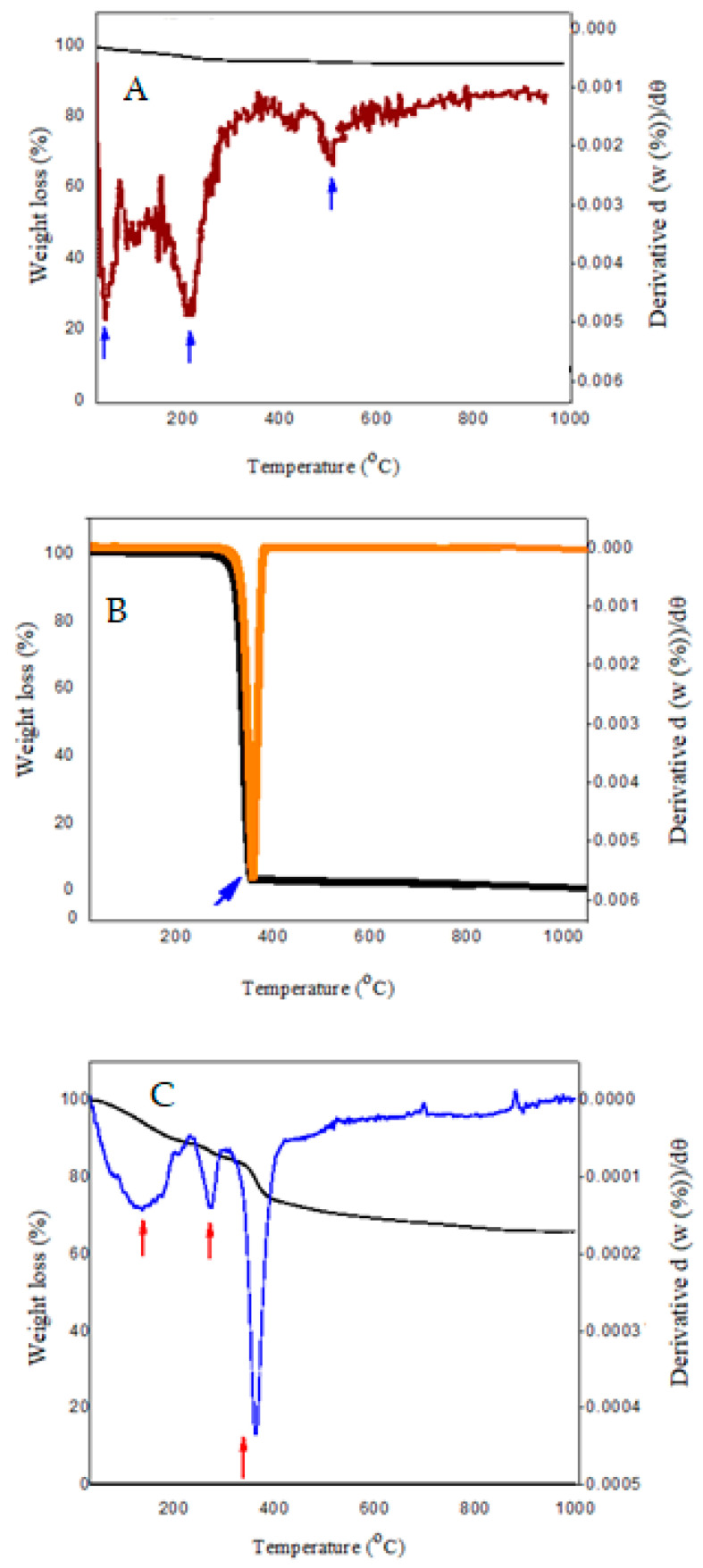
TGA/DTG thermograms of (**A**) pure Fe_3_O_4_; (**B**) net PEG; and (**C**) Fe_3_O_4_@-PEG_SFB. **Note:** Red and blue arrows represent the sharp peaks explained in the text.

Figure 4 depicts the investigation of the TGA/DTG curves related to pure samples and Fe_3_O_4_@-PEG_SFB nanocomposites during the heating process. The weight loss was observed by increasing the temperature in the DTA/TGA. Additionally, the thermal analysis of Fe_3_O_4_ nanoparticles showed an 8.89% weight loss up to 600 °C (Figure 4A). The weight loss up to 200 °C is related to absorbed water on the surface of the nanoparticles and water evaporation in the reactant [47,51]. It could be concluded that almost 5% of this weight loss is attributed to the moisture in nanoparticles. As can be observed, the thermal stability of particles up to 200 °C was significant, and no considerable weight loss was observed in this temperature range. In contrast, 3.8% weight loss occurred above that temperature up to 600 °C. Data indicated that the Fe_3_O_4_ nanoparticles with coating possessed higher thermal stability than those without coating.

The qualitative analysis of the plots showed that polymer molecules were absorbed on the surface of Fe_3_O4 nanoparticles. The rate of polymer absorption on the nanoparticles was decreased by increasing the molecular weight. When the polymer makes hydrogen bonding with Fe_3_O_4_ nanoparticles, the end of the polymer chain makes Brownian movement which prevents the absorption of other polymeric chains into the nanoparticles. Consequently, the weight of the polymer absorbed on the surface is higher.

The thermal decomposition curves of Fe_3_O_4_@PEG_SFB show that decomposition starts at 190 °C, and weight loss was observed three times for Fe_3_O_4_@PEG. The first was due to PEG degradation. According to the reactive nature of the hydroxyl groups (−OH) contained in the molecular structure of PEG, the −OH decomposes due to thermal instability when the temperature is steadily increased. In other words, PEG starts burning, and at this temperature, all branches are burnt. According to the diagram, when the temperature reaches 190 °C, a thermal release peak occurs, and the hydroxyl groups are thermally decomposed into reactive radicals and break off in large numbers. As the temperature continues to rise, the TGA curve shows that the heat break value begins to fall. Complete degradation of PEG in the Fe_3_O_4_@PEG_SFB occurred at 380 °C. This weight loss was in good agreement with the removal of residual carbon. According to the TGA results, the mass loss of Fe_3_O_4_@-PEG_SFB is faster than pure PEG. This means that in the coated sample, due to the creation of cross-linked chains in the PEG, the mass loss is less [52,53]. The last weight loss is due to the following reasons: (a) the transformation of Fe_3_O_4_ in the presence of graphite remaining from the decomposition of PEG branches. (b) the agglomeration of particles. In addition, the presence of a small peak around 700 °C can be attributed to the decomposition of Fe_3_O_4_ to other compounds such as α–ferric oxide [54]. In this stage, the remaining amount of hydroxide in the network of nanoparticles starts evaporation in this temperature range and causes weight loss in the curve of TGA. These results show that the coated nanoparticles have higher thermal stability compared to their non-coated counterpart. Additionally, the different thermal behavior of these samples confirms the successful surface modification of Fe_3_O_4_ nanoparticles by coating them with coating agents.

### 3.5. TEM Analysis

**Figure 5:**
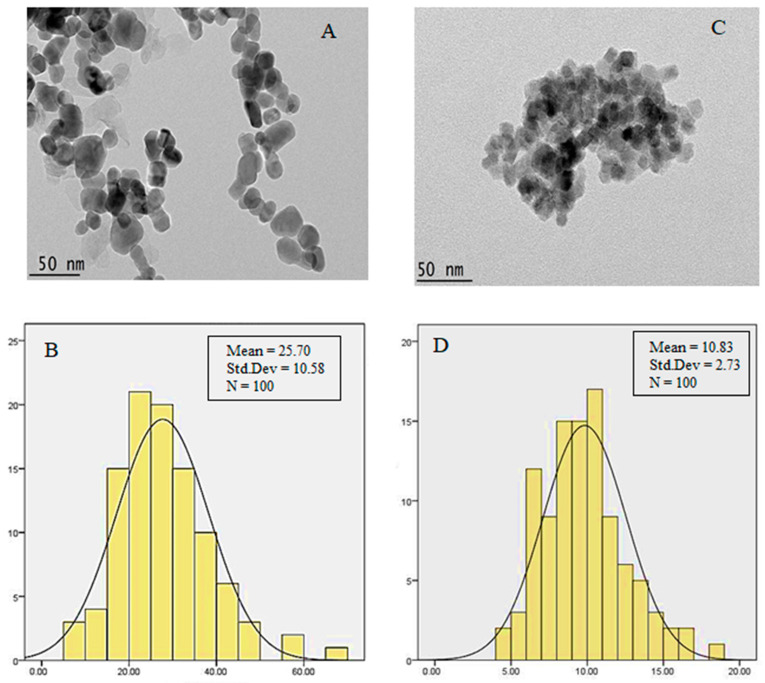
TEM micrographs of the synthesized powders (**A**) Fe_3_O_4_@-PEG-SFB; (**B**) their particle size distribution; (**C**) Fe_3_O_4_ nanoparticles; (**D**) their particle size distribution.

The microstructure details of synthesized powders were evaluated by TEM (Figure 5). As can be observed, the nanoparticles reveal a relatively uniform size distribution with spherical morphology. According to the TEM micrographs, the synthesized magnetic nanoparticles were highly agglomerated, and layers surrounded the Fe_3_O_4_ nanoparticles. This high agglomeration was due to the increase in attraction forces between the particles resulting from the increase in their ratio of surface to volume. In addition, the core-shell structure of stabilized nanoparticles with PEG is separated, and therefore the nanoparticles have more distribution due to utilizing polymer. Although a bigger particle size distribution was observed in the sample with coating, however, the surface morphology and form have remained constant with no change.

The diameter of nanoparticles was estimated using image analysis (Image J) software. The average size of nanoparticles with and without coating was found to be 25 nm and 10 nm, respectively. These sizes are proper for medical applications. As shown in Figure 5B, the dark area around the Fe_3_O_4_ nanoparticles was intensified with coated agents, clearly indicating the coating process. All the nanoparticles were covered with a PEG shell, but as shown, the coating was not completely uniform throughout the entire length of the nanoparticles.

This quantitative analysis, together with qualitative analysis using micrographs of these two samples, indicates that there is a significant difference between the two synthesized nanoparticles. According to this image, the diameter of particles was increased in the coated Fe_3_O_4,_ which indicates the successful coating of nanoparticles in the Fe3O_4_ core in a similar magnification of 40,000×. In addition, the comparison between sections (A) and (C) in Figure 5 showed that the coating of Fe_3_O_4_ did not cause accumulation and abnormal agglomeration of nanoparticles.

**Figure 6:**
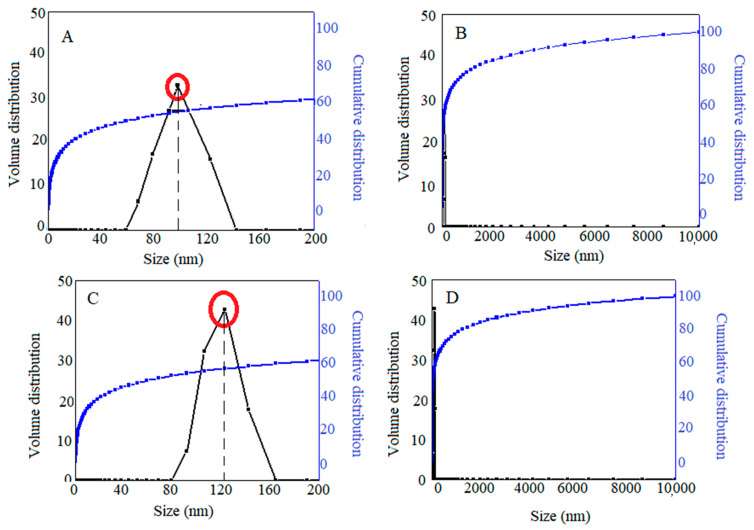
Particle size, relative of the (**A**) Fe_3_O_4_ nanoparticles; and (**C**) Fe_3_O_4_@-PEG-SFB nanocomposite; and cumulative distributions of the (**B**) Fe_3_O_4_ nanoparticles; and (**D**) Fe_3_O_4_@-PEG-SFB nanocomposite. **Note:** The red circle indicate the hydrodynamic size of nanoparticles expressed in the text.

To compare the particle size distribution and the average particle size of initial Fe_3_O_4_ particles with that of Fe_3_O_4_ particles coated with a polymer and anticancer drug, hydrodynamic particle sizes were adopted by dynamic light scattering (DLS). As can be observed in Figure 6A,B, the hydrodynamic size of initial Fe_3_O_4_ and Fe_3_O_4_ nanoparticles coated with polymer and drug was found to be 98 nm (63%) and 124 nm (62%) with a narrow size distribution, respectively. The particle size results of Fe_3_O_4_ nanoparticles in this study were in agreement with previous reports. It can be seen that the average particle size after drug loading was slightly increased. This could be attributed to the interaction of surfactant branches and the depletion phenomenon that lead to aggregation.

### 3.6. In vitro Drug Release

**Figure 7:**
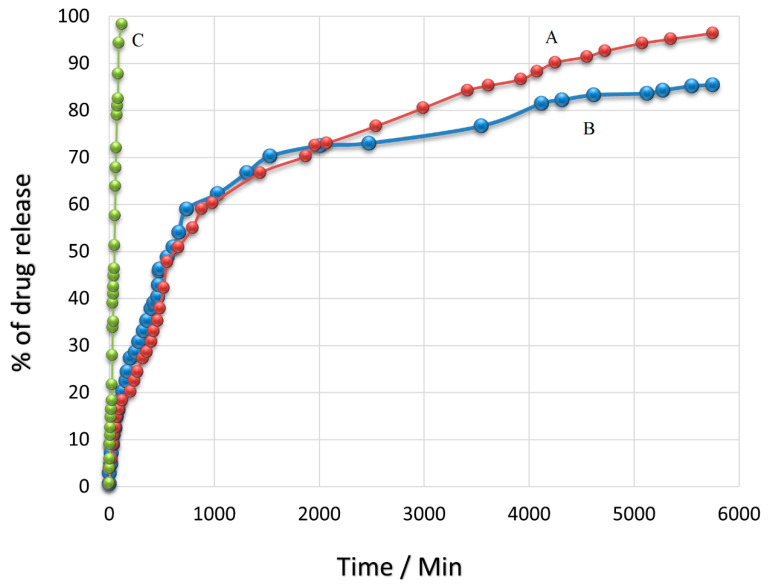
Sorafenib drug release from Fe_3_O_4_@PEG_SFB into PBS at (A) pH 4.8; (B) pH 7.4 and (C) free sorafenib.

Figure 7 shows the release profile of the SFB drug in the time intervals of 6 h for 4 days (96 h) in PBS media at a pH similar to human plasma (7.4) and cancerous areas (4.8). As can be observed, this profile is comprised of two sections; in the first section, a significant drug release of around 49% for pH = 4.8 and 46% for pH = 7.4 was observed from the beginning of the test up to the first 8 h. In the second section, more sustained drug release was observed in the time interval of 8 to 96 h and reached 96% and 85 %, which were related to pH = 4.8 and pH = 7.4, respectively. The results of the SFB drug release showed that the release was in a burst manner from PEG@Fe_3_O_4_ nanoparticles such that more than 45% of the loaded drug was released within just at first 8 h in both pH environments. Because the SFB drug can be loaded on the surface or inside the nanoparticles during the loading process on Fe_3_O_4_@PEG, it seems that in the first time interval, a high rate of drug release could be due to the drug release from the surface of the nanoparticles. After that, the polymeric nanoparticles start absorbing the water and become hydrolyzed in water. Consequently, the release of the drug, which was embedded in the structure of nanoparticles, was decreased. One of the main reasons for this decrease in drug release rate could be ascribed to the interference of PEG branches in the SFB release. Additionally, the drug needed more time to pass the surface layers. Thus, drug release at a slow rate was observed in this stage, such that around 46% of the drug was released within 88 h in the second section. In this stage, drug release was found to be uniform with a slow rate and relatively stable. In general, in this project, the SFB drug release rate decreased with the passage of time. This was due to the increase in carboxyl groups of PEG which interacts with those in SFB and prevents the exiting of SFB through polymeric branches. The results obtained in the present study were in good agreement with previous reports. A study by Varshosaz et al. [55] used different methods of loading for SFB on chitosan nanoparticles conjugated with retinoic acid. Their results showed that the size of nanoparticles was 286 nm, and the percentage of drug loading was 43%. In contrast, the synthesis method of nanoparticles and SFB loading on them in the present study led to a smaller particle size of around 100 nm and a higher percentage of loading of around 61%. In addition, Hadavand Mirzaie et al. [56] could obtain nanoparticles with a size of 150 nm with a lower percentage of drug loading compared to this study. Atabi et al. [57] have used a different method for loading SFB on PEG nanoparticles. Their findings showed a percentage of loading of around 40%. The method of synthesis for Fe_3_O_4_ nanoparticles and drug loading was optimized in this study in a way that the lowest particle size and the highest amount of drug loading were achieved. As can be seen, the SFB drug release was controlled in this system such that 96% of the total loaded drug was released continuously and sustainably within 120 h. This sustained drug release can lead to better drug efficacy and activity against cancer cells in the time intervals of drug release.

In addition, drug release studies of free SFB were carried out. The graph (Figure 7C) depicted that the release of free sorafenib was complete within 2 h, while sorafenib-incorporating nanoparticles exhibited a relatively improved delayed-release effect. Sorafenib-incorporating nanoparticles released more slowly than sorafenib alone. Fe_3_O_4_@-PEG-SFB exhibited a sustained release pattern, while free SFB showed more than 98% drug release within 2 h. The developed drug delivery system was able to sustain the drug release over 96 h; however, the free drug was released in 2 h. The results unequivocally vouch for the sustained and controlled drug release behavior of the developed drug delivery system [58,59]. In comparison with nanocarrier and nanoparticle, there is a significant reduction in cell viability compared to the nanocarrier treated alone. The nanocarrier shows minimum cytotoxicity at different concentrations compared to the nanoparticles.

### 3.7. In Vitro Bioassay and Cytotoxicity Studies

The toxicity level of SFB accompanied by PEG-Fe_3_O_4_ nanoparticles, free SFB, and SFB-containing Fe_3_O_4_-coated PEG on HepG2 and 3T3 cell lines was analyzed by conducting a cell viability assay. For this purpose, two types of cells, including hepatocellular carcinoma cells (HepG2) and normal human fibroblast (3T3) (both purchased from ATCC (Manassas, VA, USA). The Dulbecco’s Modified Eagle Medium (DMEM) (Nacalai Tesque, Kyoto, Japan) supplemented with 10% fetal bovine albumin (Sigma-Aldrich, Burlington, MA, USA), 1% antibiotics containing 10,000 units/mL penicillin and 10,000 μg/ mL streptomycin (Nacalai Tesque, Kyoto, Japan) was used to grow all the cells. Then, the humidified 5% carbon dioxide at 37 °C was used for the incubation and maintenance of cells. In the next stage, cell layers were harvested using 0.25% trypsin/1mM-EDTA (Nacalai Tesque, Kyoto, Japan). This was followed by cell seeding in 96-well tissue culture plates at 1.0 × 104 cells/well for 24 h in an incubator to attain 80% confluence for the treatment. Then the cytotoxicity and cell viability were determined by carrying out the methyl thiazole tetrazolium (MTT)-based assay. The compound was dissolved in dimethyl sulfoxide (0.1 %) and DMEM with a ratio of 1:1 to prepare the stock solutions. Then, the cells were treated with Fe, Fe_3_O_4_@PEG, free sorafenib, and Fe3O4@PEG-SFB. Finally, different concentrations in the range of 1.25 to 100 μg/mL were produced by further dilution of the mixture in the comparable media.

Upon the cell attachment to the respective walls after 24 h, the tested compounds were added to obtain a final volume of 100 μL well. Then, the addition of 10 μL of MTT solutions (5 mg/mL in PBS) and further incubation for 3 h is conducted after 72 h of incubation before aspiration. The purple formazan salt was dissolved by the addition of 100 μL of dimethyl sulfoxide per well at room temperature. The intensity of the purple formazan solution reflects cell growth. This intensity was measured at a wavelength of 570 nm utilizing a microplate reader (Biotek LE800, Winooski, VT, USA).

All the cytotoxicity assays were carried out in triplicates, and the standard deviations were calculated and incorporated in the respective bar graphs. The IC50 was calculated by plotting the x-y axis in which the values of the x-axis were converted to their corresponding log values, and a nonlinear regression (curve fit) was taken to obtain straight line equation fit, y = ax + b.

#### 3.7.1. Cellular Viability in 3T3 Fibroblast Cells

The first step in the evaluation of cytotoxicity is the MTT biocompatibility test which is an inexpensive and easy way to understand the toxicity of samples. This method is easier than other in vivo tests and faces no ethical obstacles. The key issue here is the control of environmental conditions because the cells are highly sensitive to environmental conditions, and changes can have false positive responses related to the increased toxicity of the material. In addition, it is the most available and simplest method among other different methods for the evaluation of toxicity. In this method, the MTT solution is added to insoluble formazan, which depends on the mitochondrial respiration of live cells. In this section, the results of the MTT assay for coated and uncoated nanoparticles on the two cell lines of HepG2 and 3T3. These two cell lines were used in the present study due to their wide application in the treatment of cancer cells [60].

**Figure 8:**
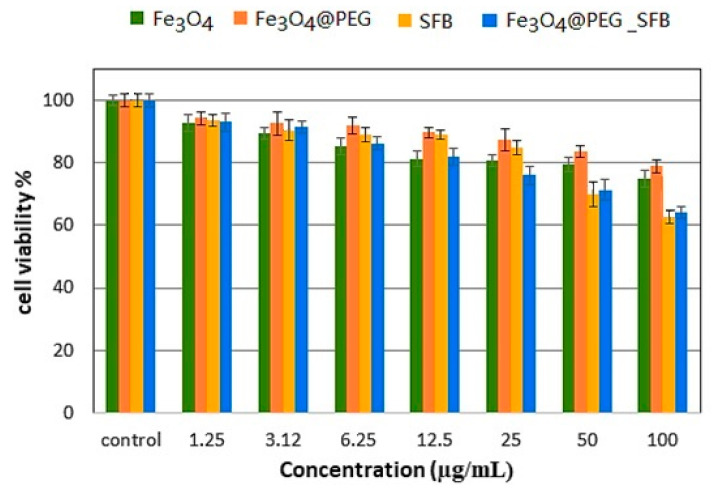
Cell viability of Fe_3_O_4_, free SFB, Fe_3_O_4_@PEG, Fe_3_O_4_@PEG_SFB against healthy 3T3 cells at 72 h. **Note:** Results were calculated as mean ± standard deviation for N = 3 independent experiments.

The percentage cell viability (the cytotoxic effect) in the time intervals of 24, 48, and 72 h incubation with various gradient concentrations of the samples (Fe_3_O_4_, Fe_3_O_4_@PEG, free SFB, and Fe_3_O_4_@PEG_SFB) treated with 3T3 cells compared with control cells is depicted in Figure 8. It was observed that all of the samples revealed an 80 % cell viability in the range of concentration for Fe_3_O_4_ (1.25–50µg), Fe_3_O_4_@PEG (1.25–100µg), free SFB (1.25–25µg), and Fe_3_O_4_@PEG_SFB (1.25–12.5µg) and the increase in concentration from 0 to 100 µg caused an increase in cytotoxicity and subsequently cell death. As can be observed, after 72 h of treatment, PEG@Fe_3_O_4_ nanoparticles were not toxic to a 3T3 cell line tested using various doses, which was in agreement with a previous report [61]. The presence of PEG brought stability and better biocompatibility to these nanoparticles and prevented toxic and pharmacokinetic effects of nanoparticles resulting from their interactions with cells or biological proteins. This suggests that the designed anticancer nanoparticle formulation is biocompatible with normal cells and would be very useful for targeting cancer cells without damaging/harming normal tissues [62].

#### 3.7.2. Evaluation of Cell Viability against HepG2

**Figure 9:**
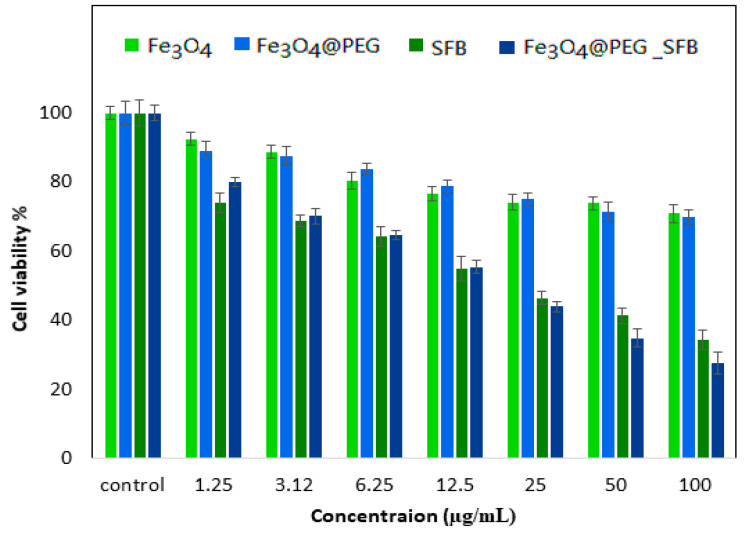
Cell viability of Fe_3_O_4_, free SFB, Fe_3_O_4_@PEG, Fe_3_O_4_@PEG_SFB against HepG2 cells at 72 h. Results were calculated as mean ± standard deviation for N = 3 independent experiments.

The MTT results on HepG2 cell lines incubated with Fe_3_O_4_, Fe_3_O_4_@PEG, free SFB, and Fe_3_O_4_@PEG-SFB samples with different concentrations in three-time intervals of 24 to 72 h are shown in Figure 9. As can be seen, the cell viability was decreased by increasing the exposure time of drug-containing nanoparticles with cancer cells. The SFB drug release from the polymeric network was increased by increasing exposure time. In addition, the highest suppressing effect on cell growth was found for Fe_3_O_4_ nanoparticles, among others, in the targeting sample. As can be observed, the nanoparticles with and without coating from the concentration of 1.25-25µg did not have a significant toxic effect on liver HepG2 cells. However, in similar time intervals, drug-containing nanoparticles were more effective in preventing the growth of cancer lines compared to free drugs. Based on the obtained results, IC_50_ values at 72 h for treatment with carriers of Fe_3_O_4_@PEG-SFB nanoparticles and free SFB against liver HepG2 cells were found to be 15.78 μg/mL and 24.48 μg/mL (*p* < 0.05). The percentage of the drug loading (81%) for Fe_3_O_4_@PEG-SFB was used to determine IC_50_. The cytotoxic effect of the drug was significantly increased by increasing the concentration from 0 to 100 μg/mL, which led to cell death. A significant difference was observed for Fe_3_O_4_@PEG_SFB nanocomposite compared to other samples at concentrations of 1.25–100 μg/mL with (*p* values of < 0.05).

Statistical analysis was determined using software, SPSS and ANOVA, and Duncan’s Multiple Range Test. At a concentration of 12.5–100 μg/mL, SFB was significantly different from the empty nanocarrier (*p*-value < 0.05). The samples of free SFB and Fe_3_O_4_@PEG_SFB showed a dose-dependent anticancer effect against the cell line. The IC_50_ of all the samples is tabulated in Table 1. The IC_50_ indicated a better anticancer effect for synthesized nanoparticles than the drug in their free forms. Aljarrah et al. have synthesized iron nanoparticles and have shown that these nanoparticles alone cannot impede human breast cancer (MCF-7) cell growth. Additionally, their MTT results have revealed that the cell viability was decreased by increasing the concentration. In addition, the cytotoxic effect of the pure drug and loaded drug on magnetic nanoparticles in a static magnetic field and different concentrations of 0.1, 0.2, 0.3, and 0.5 M revealed more toxicity for the loaded drug than that of the pure counterpart. The results obtained for PEG-PLGA/SFB nanoparticles showed more toxicity compared to pure SFB without coating with comparable SFB concentration. The reason for this phenomenon is the higher absorption of SFB drug to MCF-7 cancer cells due to their smaller size. Surface modification can be used to increase the biocompatibility of these magnetic nanoparticles using different surface coatings such as PEG. The presence of PEG brought stability and better biocompatibility to these nanoparticles and prevented toxic and pharmacokinetic effects of nanoparticles resulting from their interactions with cells or biological proteins. Consequently, the biocompatibility of magnetic nanoparticles was increased. The evaluation of FTIR spectra and XRD confirmed the capability and biocompatibility of PEG@Fe_3_O_4_ nanoparticles to infiltrate into the cells.

## 4. Conclusions

In the present study, Fe_3_O_4_ nanoparticles were synthesized. Then, the PEG and SFB were successfully coated on the Fe_3_O_4_ NPs. The presence of magnetite core, polymer, and drug as a shell in the Fe_3_O_4_@-PEG-SFB was demonstrated by XRD analysis. Additionally, the crystalline structure of magnetite and modified magnetite was well-matched with the JCPDS card. The particle size distribution and morphology of synthesized nanoparticles were analyzed by TEM and DLS analysis, and it was found that the average nanoparticle sizes of 25 nm and 10 nm for Fe_3_O_4_@-PEG-SFB and Fe_3_O_4_, respectively. The magnetic properties of synthesized nanoparticles were evaluated by VSM analysis, and the saturation magnetism of Fe_3_O_4_@-PEG-SFB was measured at 37 emu/g. In addition, the magnetic coercivity and magnetic hysteresis were found around zero, indicating the superparamagnetic characteristic of nanoparticles. Furthermore, the surface bonding and surface modification of nanoparticles by functional polymeric groups were evaluated by FTIR analysis, and it was confirmed that the surface of nanoparticles was successfully coated with carbon branches. A sustained drug delivery of sorafenib was observed in which 96% of the present drug was sustainably and continuously within 96 h. The drug released profile favors the nanoparticle’s treatment time with significant IC_50_ in cell cytotoxicity. It was found that Fe_3_O_4_@-PEG-SFB was biocompatible on 3T3 cells while showing cytotoxicity toward HepG2 cancer cells. In in vitro bioassay, the study indicated the drug delivery system in this study could enhance the efficiency of anticancer drugs in inhibiting the growth of cancer cells.

## Figures and Tables

**Table 1:** IC_50_ value (the half-maximal inhibitory concentration) for Fe_3_O_4_, free SFB, Fe_3_O_4_@PEG, and Fe_3_O_4_@PEG_SFB samples tested on 3T3 and HepG2 cell lines.

Nanoparticles IC_50_ (μg/mL)	3T3 Fibroblast Cell	HepG2 Cells
Fe_3_O_4_	No cytotoxicity	No cytotoxicity
Fe_3_O_4_@PEG	No cytotoxicity	No cytotoxicity
Fe_3_O_4_@PEG_SFB	No cytotoxicity	No cytotoxicity
SFB	No cytotoxicity	24.48

## Data Availability

Not applicable.
